# Subjective Cognitive Decline as a Preclinical Marker for Alzheimer's Disease: The Challenge of Stability Over Time

**DOI:** 10.3389/fnagi.2017.00377

**Published:** 2017-11-21

**Authors:** Marina Ávila-Villanueva, Miguel A. Fernández-Blázquez

**Affiliations:** Alzheimer Disease Research Unit, CIEN Foundation, Carlos III Institute of Health, Queen Sofía Foundation Alzheimer Center, Madrid, Spain

**Keywords:** aging, Alzheimer disease, cognitive symptoms, dementia, stability, subjective cognitive decline

Subjective Cognitive Decline (SCD) refers to a self-experienced persistent decline in cognitive abilities in comparison with a prior normal status and independent of the objective performance on neuropsychological tests (Jessen et al., 2014). It has been proposed that SCD might appear at the end of the preclinical phase of Alzheimer's Disease (AD) even in the absence of significant objective impairment detectable on standardized neuropsychological assessment (Rabin et al., 2017). This fact explains why SCD is gaining increased prominence in neurodegenerative research as a potential marker for future Mild Cognitive Impairment (MCI) due to AD. Nevertheless, in our opinion SCD has to face up to a challenge in order to aspire to become a reliable marker of preclinical AD. This challenge is related to the temporal stability of self-reported complaints over time. This manuscript describes this challenge.

SCD is considered a sign of preclinical AD that occurs even before objective cognitive impairment appears (Figure 1). A recent meta-analysis has revealed that about 25% of cognitively healthy older adults who report SCD will develop MCI due to AD in the next 4 years (Mitchell et al., 2014). In addition, these individuals have two-fold risk of progression to dementia during a 5-year follow-up period.

**Figure 1:**
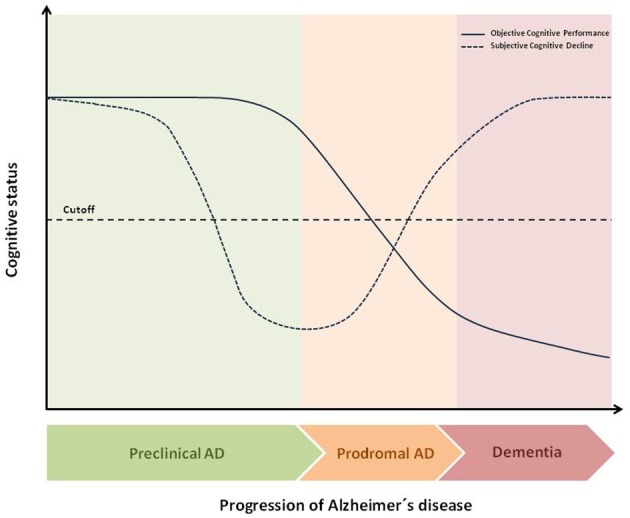
Theoretical temporal dynamic of objective and subjective cognitive decline throughout AD continuum. The figure shows the hypothetical differences, during the transition from preclinical AD to dementia, between Subjective Cognitive Decline (SCD) and Objective Cognitive Decline (OCP). At final stages of preclinical AD, SCD is a better early marker than OCP for the transition to Mild Cognitive Impairment (MCI).As disease progresses, cognitive performance decreases and at prodromal stage, (MCI) both SCD and OCP are below cutoff. Indeed, as disease progresses SCD usually disappears leading to a deficit of self-awareness about the own disabilities namely anosognosia.

To increase the potential usefulness of SCD the international working group called Subjective Cognitive Decline Initiative (SCD-I) agreed to a common framework and research procedures to study the role of SCD as a marker of preclinical AD (Jessen et al., 2014). Following these new standards, cognitively healthy individuals who accomplish certain conditions of SCD have been probed to have four times higher risk for developing prodromal AD in just 1-year compared to those subjects without complaints (Fernández-Blázquez et al., 2016). Despite its outstanding clinical value, recently the SCD-I also pointed out some limitations of SCD when it comes to investigating this concept (Rabin et al., 2015; Molinuevo et al., 2017). These limitations could be summarized in three different blocks:

1. Terminology has not been homogeneous across studies and terms such as “subjective memory complaints,” “subjective cognitive complaints,” “subjective cognitive decline” or “subjective memory impairment” have been used interchangeably to refer to the same underlying concept. This lack of consensus on a single definition of SCD might affect to the comparison of findings from different investigations.
2. Methodology and tools to assess SCD are also heterogeneous. This includes the context in which the sample is recruited (clinical vs. community-based), the mode of administration of measures (structured interview conducted by an examiner vs. self-reported questionnaires), the cognitive domains that must be examined (memory vs. non-memory domains), the number of items to be used (one or two questions vs. scales with a large number of items), the way to respond the questions (opened questions vs. multiple choice), and the timeframe to collect data (shorter vs. longer periods of time). This heterogeneity may lead to contradictory results and needs to be carefully addressed.
3. Operational criteria and cutoffs to assess the degree of confidence in the self-report of SCD differ across studies.

This heterogeneity in definitions, in approaches for measuring SCD, and in operational criteria emphasizes the necessity of having available a shared terminology and common frameworks of evaluation. To settle these limitations the SCD-I group has proposed a number of recommendations that specifically address the currently existing limitations (Molinuevo et al., 2017). As a first step, it is important to select the most appropriate measures that should be related to the characteristics of the target population. Cognitive complaints may have different implications depending on the research context where they are gathered. For instance, the concerns on SCD in clinical samples may be higher than in community-dwelling individuals. Moreover, it would be suitable to rely on tools with adequate psychometric properties for the reference population. As a second step, the SCD's measures must have appropriate content coverage with regards to the target population. In this way, all items should be well-written and understandable, avoiding double meanings and inquiring for difficulties often found in daily life. In a third step, measures should explore different cognitive domains (such as attention and executive function) because the earliest symptoms of AD may affect beyond memory. In the fourth step, the response options for all measures should be selected depending on the study aims. When the purpose is to distinguish between groups, dichotomous items may be enough. However, if we are interested in monitoring the change of SCD over time, ordinal response options should be preferred. Finally, another critical point is to determine the reference period of time in which we want to examine the SCD. Generally, inquiring over short periods of time (no longer than 1 year) allow us to reduce problems with retrospective recall or estimation of SCD. Nevertheless, we can of course ask for longer periods if we want to study the progression of SCD throughout the lifetime.

There is however a crucial limitation that deserves particular attention and has not been conveniently addressed yet, namely, the stability of complaints over time. In psychometric terms, when we are measuring subjective variables like SCD we are actually obtaining two different types of information: (i) the construct of interest (i.e., SCD in our case); and (ii) errors of measurement which comprise the error variance and include information regarding other irrelevant constructs (e.g., depressive symptoms associated with a particular complaint, personality traits, etc.) as well as short-term fluctuations due to shifts in self-perception itself. Thus, when repeated subjective measurements are collected from an individual the scores on two different occasions may be quite different (Nesselroade and Salthouse, 2004). If this were the case in the majority of subjects, the subjective variable would lack internal consistency. In other words, if two longitudinal measures are quite different and they do not converge, which one is a better assessment of the individual? This lack of temporal stability, which can affect preferably to subjective measures rather than objective performance, represents an important bias to investigation. Thus, if a construct do not probe to be stable enough over time it should not be considered as a target for research. Only demonstrating that SCD is a robust and stable concept it could become a reliable preclinical marker for AD.

To probe the stability of SCD, we propose to harmonize a protocol to gather all relevant information about cognitive complaints and to compare longitudinally the responses of the individuals. We specifically suggest collecting information combining different approaches to ensure the greatest possible internal consistency. An interesting position would be to gather self-perceived data using two procedures: a face-to-face interview with a healthcare professional and a self-administered questionnaire of cognitive complaints. It should be desirable combining both open-ended questions and structured scales in order to measure different features of SCD. The use of questionnaires is highly recommended to quantify SCD somehow and to monitor the progression of cognitive complaints over time. Additionally, a multiple choice approach should vary from dichotomic to ordinal Likert-type scales to grasp the dimensionality of SCD in the best way possible.

Regarding the content of SCD to be collected, we propose to measure a set of clinical details of the self-experienced cognitive decline. Variables such as age at onset, time of progression, memory performance compared to other people, concerns associated with SCD, and frequency of particular cognitive complaints are relevant data that must be carefully obtained. Moreover, SCD should not be examined in isolation to examine the effect of complaints upon AD. Demographic variables such as age, gender, and education, as well as medical and lifestyle variables can be gathered very easily by means of a survey. These variables have the greatest interest due to their possible implication in the expression of SCD. Objective cognitive performance and diagnosis are also critical to establish the current stage of an individual in the AD continuum and the relationship between SCD and risk of developing MCI and AD. Finally, neuropsychiatric variables should be collected as well because of their mediator role between SCD and cognitive decline.

In conclusion, SCD has been proposed to appear at the end of the preclinical phase of AD even in the absence of significant objective impairment (Rabin et al., 2017). As a result, the research of SCD as a potential marker for future MCI is increasing. In any event, this construct must deal with some limitations that have been already pointed out by the SCD-I (Molinuevo et al., 2017). However, in our opinion SCD has to face up to another challenge not brought to the table so far that is related to the temporal stability of complaints over time. If SCD does not probe to have enough internal consistency, then this construct cannot be considered as a reliable marker of preclinical AD. Future directions to study the relationship between SCD and conversion rate to MCI may involve the analysis of some covariates such as age, education, depression, anxiety and presence of ApoE ε4 allele.

## Author contributions

MÁ-V and MF-B are responsible for the conceptualization, reviewing the literature, and critically editing the manuscript. Both authors approve the submitted version of the manuscript and are accountable for the accuracy and integrity of the work.

### Conflict of interest statement

The authors declare that the research was conducted in the absence of any commercial or financial relationships that could be construed as a potential conflict of interest.
